# Pubertal timing and tempo and body mass index trajectories: investigating the confounding role of childhood body mass index

**DOI:** 10.1093/aje/kwaf063

**Published:** 2025-03-21

**Authors:** Anne Gaml-Sørensen, Nis Brix, Andreas Ernst, Lea Lykke Harrits Lunddorf, Onyebuchi A Arah, Katrine Strandberg-Larsen, Cecilia Høst Ramlau-Hansen

**Affiliations:** Department of Public Health, Research Unit for Epidemiology, Aarhus University, 8000 Aarhus C., Denmark; Department of Public Health, Research Unit for Epidemiology, Aarhus University, 8000 Aarhus C., Denmark; Department of Clinical Genetics, Vejle Regional Hospital, The Region of Southern Denmark, 7100 Vejle, Denmark; Department of Public Health, Research Unit for Epidemiology, Aarhus University, 8000 Aarhus C., Denmark; Department of Urology, Aarhus University Hospital, 8200 Aarhus N, Denmark; Department of Public Health, Research Unit for Epidemiology, Aarhus University, 8000 Aarhus C., Denmark; Department of Public Health, Research Unit for Epidemiology, Aarhus University, 8000 Aarhus C., Denmark; Department of Epidemiology, Fielding School of Public Health, University of California, Los Angeles (UCLA), Los Angeles, CA 90095, United States; Department of Statistics and Data Science, Division of Physical Sciences, UCLA College, Los Angeles, CA 90095, United States; Practical Causal Inference Lab, Fielding School of Public Health, UCLA, Los Angeles, CA 90095, United States; Department of Public Health, Section of Epidemiology, University of Copenhagen, 1353 Copenhagen K, Denmark; Department of Public Health, Research Unit for Epidemiology, Aarhus University, 8000 Aarhus C., Denmark

**Keywords:** growth, height, weight, body mass index, trajectories, puberty, Tanner stages

## Abstract

Earlier pubertal timing and faster pubertal tempo (pace of progression through puberty) might be associated with increased body mass index (BMI) later in life. In a follow-up study of 13 219 boys and girls from the Danish National Birth Cohort, we investigated the association between pubertal timing and tempo and BMI trajectories from puberty to adulthood and explored the potential confounding role of childhood BMI. Based on half-yearly information on self-reported current Tanner stages, pubertal timing and tempo were estimated using nonlinear mixed-effect growth models. In total, 136 457 height and weight measurements from 7 to 18 years were included. BMI trajectories from 11 to 18 years were fitted according to pubertal timing and tempo while adjusting for potential confounders, including childhood BMI at age 7 years. Children with earlier pubertal timing had higher and children with later pubertal timing had lower BMI trajectories from 11 to 18 years than children with average pubertal timing. After adjustment for childhood BMI, the difference disappeared in boys but persisted in girls, suggesting that earlier pubertal timing may be independently associated with later BMI in girls only. Faster pubertal tempo was associated with slightly higher BMI in young women only.

## Introduction

Children are experiencing puberty at a younger age than previously.^1^^-^^3^ This might be a contributing factor to the obesity epidemic in adulthood,^4^ as earlier puberty has been associated with increased body mass index (BMI) in adulthood in women and, to some extent, also in men.^5^^-^^8^ It is currently unsettled whether this association is confounded by increased childhood BMI,^5^^,^^9^ which might accelerate pubertal timing and is also an independent risk factor for higher adult BMI.^5^^,^^10^ A review from 2013 reported that studies exploring the potential confounding role of an increased BMI in childhood in the association between pubertal timing and higher BMI in adulthood have found conflicting results.^5^ Since then, some studies have investigated the association between pubertal timing and BMI in adulthood, with and without adjusting for childhood BMI, but results are still inconclusive (Table S1). Moreover, most studies relied only on age at menarche in girls, and only a few studies included information on boys (Table S1).

Pubertal tempo—the pace of progression through puberty—is a far less studied marker of pubertal development but may be equally important for both physical and mental health later in life.^11^ Pubertal tempo has been linked to higher BMI in a single study.^9^ The study found that fast progression through puberty was associated with higher BMI at 18 to 20 years in both men and women, an association that attenuated after adjustment for childhood BMI, indicating potential confounding by childhood BMI.^9^

In this study, we explore in a large population-based sample of young males and females whether pubertal timing and tempo are associated with BMI trajectories from puberty to adulthood, also after accounting for childhood BMI.

## Methods

This population-based cohort study is based on the Danish National Birth Cohort (DNBC),^12^ the Puberty Cohort nested within the DNBC,^13^ and linked with data on objectively measured weight and height retrieved from the Danish Children’s Database.^14^

The DNBC consists of approximately 92 000 pregnant women and their children. The pregnant women were enrolled in early pregnancy with their general practitioner from 1996 to 2002. They provided information on health and health behavior through computer-assisted telephone interviews twice during pregnancy and at 6 and 18 months postpartum. Comprehensive follow-up questionnaires, which also included questions on height and weight of the children, were sent to the families by mail or e-mail when the children were 7, 11, and 18 years old.

The Puberty Cohort consists of 22 439 children sampled from 56 641 eligible children. Eligible children were live-born singletons born from 2000 to 2003 in the DNBC, whose mothers participated in the first interview during pregnancy. In May 2012, the children invited to participate in the Puberty Cohort were sampled from 27 different exposure groups from 15 prenatal exposures, hypothesized to be causes of earlier puberty, and combined with a random sample of 8000 children.^15^ Sampling weights to account for the sampling regime have been described in detail previously.^15^ From August 2012 to January 2021, sampled children were invited to provide half-yearly information on pubertal development, height, and weight from 11.5 years until 18 years of age or full maturity. During the 11-year follow-up in the DNBC, the children also provided information on their pubertal development. When these 2 data sources were combined, 15 819 children (participation rate 70%) returned at least 1 questionnaire with information on pubertal development. To obtain the timing and tempo of pubertal development, researchers argue that at least 2 data points are needed,^16^ and therefore, we included 13 219 participants (corresponding to 59% of the invited children) with a minimum of 2 questionnaires in the final study population (Figure S1, Table S2).

### Tanner stages

Pubertal timing and tempo were measured using information on Tanner stages. The puberty questionnaires were used to collect information on the children’s current Tanner stage^1^^-^^5^ for breast and pubic development in girls^17^ and genital and pubic hair development in boys.^18^ This information was obtained using illustrations of the Tanner stages together with a short text describing each stage. The questionnaires can be accessed at http://www.dnbc.dk/data-available/puberty-follow-up.

### Height, weight, and body mass index

Height (cm) and weight (kg) were obtained from the DNBC Puberty Cohort, the 7-year, 11-year, and 18-year follow-up in the DNBC and the Children’s Database. In the DNBC, height and weight measures were self-reported by the mothers until the child was 11 years old and then by the participants themselves on 108 679 occasions for the 13 219 children during the different follow-up waves. The specific timing of measurement was recorded for the 7-year follow-up (date of measurement), for the Puberty Cohort; the 11-year follow-up timing of measurement was recorded in 4 categories (today, < 1, 1-6, > 6 months); and for the 18-year follow-up, timing of measurement was recorded in 5 categories (today, < 1, 1-6, > 6 to 12, > 12 months). We only used height and weight measured within the past year, and the age at the height and weight measurement was corrected for this information (<1: 0.5 month, 1-6: 3 months, > 6 to 12: 9 months, or specific measurement date for the 7-year follow-up).

The Children’s Database was established in April 2009 and holds information on height and weight of Danish children obtained during preventive health visits at school nurses and general practitioners.^14^ The children were measured in light clothes and without shoes using a stadiometer according to guidelines. Reporting has been mandatory since December 2011. In July 2017, height and weight data were extracted for all children invited for the Puberty Cohort. The specific date of measurement was recorded. In total, height and weight were measured on 27 778 occasions for the children in the study population.

Therefore, a total of 136 457 height and weight measurements were available for the 13 219 children included in the analyses, with a median of 10 (25th percentile and 75th percentile, 7 and 13) height and weight measures per child (Figure S1). BMI in kg/m^2^ was derived as “weight in kg” divided by “height in meters squared.”

### Covariates

Potential confounders for the association between pubertal timing and tempo and BMI trajectories were identified based on a directed acyclic graph^19^ (Figure S2) and included maternal age at menarche, maternal prepregnancy BMI, first-trimester maternal smoking, highest educational level of the parents, birth weight, and childhood BMI at 7 years. The highest educational level of parents was categorized according to the International Standard Classification of Occupation and Education codes (ISCO-88 and ISCED) and obtained from the first interview in the DNBC, together with information on maternal age at menarche, prepregnancy BMI, and first-trimester maternal smoking. Childhood BMI was obtained by parental self-report from the 7-year follow-up in the DNBC, and birth weight was obtained from the Danish Medical Birth Register.^20^ Covariates were categorized or kept continuous, as shown in Table 1 and Table 2.

**Table 1:** Characteristics by timing of puberty, the Puberty Cohort, the Danish National Birth Cohort, Denmark, 2000-2021.

	**Timing of genital development in boys, y**			**Timing of breast development in girls, y**			
**Pubertal timing**	**Early**	**Average**	**Late**	**Early**	**Average**	**Late**	**Missing, %**
n, %	940 (15.0)	4388 (70.0)	940 (15.0)	1041 (15.0)	4861 (70.0)	1041 (15.0)	0
Mean (SD), y	11.4 (0.5)	13.1 (0.6)	14.9 (0.6)	10.6 (0.5)	12.3 (0.7)	14.2 (0.6)	
Pseudo-range	9.7-12.0	12.0-14.2	14.2-17.5	9.2-11.2	11.2-13.6	13.6-17.0	
Highest educational level of the parents							0.2
High-grade professional	242 (25.8)	1056 (24.2)	223 (23.8)	207 (19.9)	1141 (23.5)	274 (26.3)	
Low-grade professional	<273^a^ (<29.0)	1487 (34.0)	<345^a^ (<36.7)	<356^a^ (<34.2)	1651 (34.0)	<361^a^ (<34.7)	
Skilled worker	268 (28.6)	1173 (26.8)	260 (27.7)	296 (28.5)	1294 (26.6)	280 (26.9)	
Unskilled worker	129 (13.8)	555 (12.7)	88 (9.4)	160 (15.4)	635 (13.1)	103 (9.9)	
Student and economically inactive	28 (3.0)	101 (2.3)	24 (2.6)	22 (2.1)	135 (2.8)	23 (2.2)	
Maternal age at menarche							0.8
Earlier than peers	285 (30.5)	1095 (25.2)	185 (19.8)	368 (35.6)	1252 (25.9)	155 (15.0)	
Same time as peers	521 (55.7)	2493 (57.3)	562 (60.3)	559 (54.1)	2765 (57.3)	607 (58.9)	
Later than peers	129 (13.8)	762 (17.5)	185 (19.8)	107 (10.3)	809 (16.8)	268 (26.0)	
Maternal prepregnancy BMI							1.5
< 18.5	39 (4.2)	306 (7.1)	57 (6.2)	68 (6.7)	311 (6.5)	88 (8.5)	
18.5 to < 25	585 (63.2)	2732 (63.1)	600 (64.8)	565 (55.4)	2931 (61.2)	733 (71.2)	
25 to < 30	203 (21.9)	863 (19.9)	177 (19.1)	255 (25.0)	1051 (21.9)	151 (14.7)	
30+	98 (10.6)	426 (9.8)	92 (9.9)	132 (12.9)	498 (10.4)	58 (5.6)	
Maternal smoking in first trimester							0.3
Nonsmoker	<634^a^ (<67.4)	3209 (73.4)	749 (79.7)	<671^a^ (<64.5)	3505 (72.4)	<816^a^ (<78.4)	
≤10 cigarettes/day	231 (24.6)	946 (21.6)	155 (16.5)	277 (26.7)	1082 (22.4)	185 (17.8)	
>10 cigarettes/day	75 (8.0)	217 (5.0)	36 (3.8)	93 (9.0)	253 (5.2)	40 (3.8)	
Birth weight,^b^ kg	3600 (624)	3581 (624)	3598 (584)	3474 (575)	3474 (564)	3466 (564)	0.4
Childhood BMI at 7 years^b^	16.1 (1.8)	15.7 (1.7)	15.6 (1.7)	16.1 (1.8)	15.6 (1.7)	14.9 (1.5)	26

Abbreviations: BMI, body mass index; SD, standard deviation.

Due to rounding of percentages, numbers may not add up to 100%.

a It is not allowed due to local data regulations to report smaller numbers than 5, including missing data. Therefore, some numbers have been rounded up or down to mask the numbers smaller than 5.

b Mean (SD).

**Table 2:** Characteristics by tempo of puberty, the Puberty Cohort, the Danish National Birth Cohort, Denmark, 2000-2021.

	**Tempo of genital development in boys** **(Tanner stages per year)**			**Tempo of breast development in girls** **(Tanner stages per year)**			
**Pubertal tempo**	**Fast**	**Average**	**Slow**	**Fast**	**Average**	**Slow**	**Missing, %**
n, %	940 (15.0)	4388 (70.0)	940 (15.0)	1041 (15.0)	4861 (70.0)	1041 (15.0)	0
Mean (Tanner stages per year, SD)	0.4 (0.2)	0.9 (0.2)	1.4 (0.1)	0.3 (0.1)	0.7 (0.2)	1.1 (0.1)	
Pseudo-range	–0.7 to 0.6	0.6-1.2	1.2-2.0	0.0-0.4	0.4-1.0	1.0-1.6	
Highest educational level of the parents							0.2
High-grade professional	243 (25.9)	1035 (23.7)	243 (25.9)	220 (21.2)	1165 (24.0)	237 (22.8)	
Low-grade professional	<283^a^ (<30.1)	1473 (33.7)	<349^a^ (<37.1)	<368^a^ (<35.4)	1638 (33.7)	362 (34.8)	
Skilled worker	258 (27.5)	1202 (27.5)	241 (25.7)	304 (29.3)	1284 (26.4)	282 (27.1)	
Unskilled worker	128 (13.7)	555 (12.7)	89 (9.5)	128 (12.3)	634 (13.1)	136 (13.1)	
Student and economically inactive	28 (3.0)	107 (2.4)	18 (1.9)	21 (2.0)	135 (2.8)	24 (2.3)	
Maternal age at menarche							0.8
Earlier than peers	255 (27.2)	1093 (25.1)	217 (23.3)	284 (27.5)	1225 (25.4)	266 (25.8)	
Same time as peers	<532^a^ (<56.6)	2497 (57.4)	551 (59.1)	565 (54.7)	2769 (57.4)	597 (57.8)	
Later than peers	153 (16.3)	759 (17.5)	164 (17.6)	184 (17.8)	830 (17.2)	170 (16.5)	
Maternal prepregnancy BMI							1.5
< 18.5	60 (6.5)	284 (6.6)	58 (6.3)	62 (6.1)	334 (7.0)	71 (6.9)	
18.5 to < 25	560 (60.6)	2762 (63.8)	595 (64.4)	603 (58.9)	3022 (63.1)	604 (58.9)	
25 to < 30	205 (22.2)	862 (19.9)	176 (19.0)	242 (23.6)	970 (20.2)	245 (23.9)	
30+	99 (10.7)	422 (9.7)	95 (10.3)	117 (11.4)	465 (9.7)	106 (10.3)	
Maternal smoking in first trimester							0.3
Nonsmoker	<684^a^ (<72.8)	3165 (72.3)	<747^a^ (<79.5)	<750^a^ (<72.0)	3475 (71.8)	<768^a^ (<73.8)	
≤10 cigarettes/day	208 (22.2)	966 (22.1)	158 (16.9)	220 (21.2)	1099 (22.7)	225 (21.7)	
>10 cigarettes/day	48 (5.1)	245 (5.6)	35 (3.7)	71 (6.8)	267 (5.5)	48 (4.6)	
Birth weight,^b^ kg	3579 (633)	3592 (617)	3568 (609)	3453 (556)	3474 (565)	3485 (575)	0.4
Childhood BMI at 7 years^b^	15.8 (1.8)	15.7 (1.7)	15.7 (1.7)	15.6 (1.7)	15.5 (1.7)	15.6 (1.8)	26

Abbreviations: BMI, body mass index; SD, standard deviation.

Due to rounding of percentages, numbers may not add up to 100%.

a It is not allowed due to local data regulations to report smaller numbers than 5, including missing data. Therefore, some numbers have been rounded up or down to mask the numbers smaller than 5.

b Mean (SD).

### Statistical analysis

Pubertal timing was derived from the age at mid-puberty (ie, at Tanner stage 3), and pubertal tempo was derived as the pace of progression in Tanner stages per year at mid-puberty (ie, at Tanner stage 3). We used nonlinear mixed-effect models to estimate the individual timing and tempo of each of the 4 Tanner traits (breast development in girls, genital development in boys, and pubic hair in both sexes) using the *menl* package in Stata. These models have been described in detail by Marceau et al^21^ and by Lunddorf et al^22^ but are briefly described here. Nonlinear mixed-effect models were fitted for each of the 4 Tanner traits with *timing* and *tempo* as the only 2 model parameters (each with a fixed effect and a random term). Based on these models, the individual pubertal timing and tempo were derived using the Lindstrom-Bates estimation method, which is similar to the best linear unbiased prediction in linear mixed-effect models. These individual *timing* and *tempo* estimates describe that individual’s logistic (ie, S-shaped) Tanner trajectory (eg, for Tanner breast development). Pubertal timing was then categorized as earlier (<15th percentile), average (15th-85th percentile, reference), or later (>85th percentile) timing. Similarly, pubertal tempo was categorized as slower (<15th percentile), average (15th-85th percentile, reference), or faster (>85th percentile) tempo. All percentiles in tables and figures are presented as pseudo-percentiles, calculated as the average of the 5 values nearest the actual percentile to comply with local regulations (General Data Protection Regulation, Regulation [EU], 2016/679 of May 25, 2018).

In a descriptive analysis, we modeled height, weight, and BMI trajectories from 7 to 18 years of age according to categorizations of pubertal timing and tempo for each of the Tanner traits. In the analyses, height, weight, and BMI trajectories were fitted using linear regression with robust standard errors to allow for multiple outcome measures per participant.^23^^,^^24^ Pubertal timing and tempo were included as indicator variables, and age was included as a restricted cubic spline with knots at 11.0, 12.5, 14.5, and 16.5 years, while allowing for interaction with pubertal timing and tempo, respectively.

In the main analysis, we modeled BMI trajectories from 11 to 18 years as described above, but with age included as a restricted cubic spline with knots at 11.5, 12.5, 14.5, and 16.5 years while allowing for interaction with pubertal timing and tempo, respectively.

In subanalyses, we analyzed the associations between pubertal timing and tempo for each of the Tanner traits separately in addition to the joint effect of pubertal timing and tempo on adult BMI at 18 years using linear regression.

Although our directed acyclic graph depicted childhood BMI as a shared cause (ie, a confounder) of pubertal timing and tempo and adult BMI, we aimed to explore the confounding role of childhood BMI. Therefore, all analyses were conducted in 3 steps: (1) estimating the crude association, (2) adjusting for all potential confounding factors except childhood BMI at 7 years (partly adjusted), and (3) further adjusting for childhood BMI (fully adjusted).

Selection weights were derived to account for incomplete participation.^25^ We used a logistic regression to estimate the selection weights as the inverse probability of participation (ie, having the above information and consequently being included in the main analyses or subanalysis), given the highest educational level of parents, maternal age at menarche, maternal prepregnancy BMI, and maternal smoking during pregnancy. These weights were multiplied by the sampling weights estimated to account for the sampling regime employed in the Puberty Cohort^15^ and used to reweight all analyses, thereby making the results generalizable to the 56 641 children eligible for being invited to the Puberty Cohort. Robust standard errors were employed in all analyses to account for multiple observations per participant, as well as the use of inverse probability weights. Data management and statistical analyses were conducted in Stata 17.0 (StataCorp LLC).

## Results

In total, 12 812 (6075 boys and 6737 girls) were included in the descriptive analysis, 9520 (4609 boys and 4911 girls) were included in the main analysis, and 5381 (2111 boys and 3270 girls) were included in the subanalysis (Figure S1).

The mean (standard deviation [SD]) timing of Tanner genital stage 3 in boys was 13.1 (1.1) years, and the mean (SD) timing of Tanner breast stage 3 in girls was 12.3 (1.2) years. The mean (SD) tempo of genital development in boys was 0.9 (0.3) Tanner stages per year, and the mean (SD) tempo of breast development in girls was 0.7 (0.3) Tanner stages per year.

Children with earlier pubertal timing had lower educated parents, had mothers who had earlier age at menarche, had a higher prepregnancy BMI, and were more often smokers. Contrary, children with later pubertal timing had higher educated parents, had mothers who had later age at menarche, had a lower prepregnancy BMI, and were more often nonsmokers (Table 1). Overall, the same pattern was observed for pubertal tempo, albeit not as strong (Table 2). Children with earlier pubertal timing had a higher mean BMI at age 7 years compared to children with average and later pubertal timing (Table 1), whereas childhood BMI did not differ across categorizations of pubertal tempo (Table 2).

### Descriptive analysis

For each of the Tanner traits, children with earlier pubertal timing had higher BMI and children with later pubertal timing had lower BMI than children with average pubertal timing throughout age 7 to 18 years (Figure S3). Boys with faster pubic hair development had a higher BMI at around 8 to 14 years of age (Figure S4f). Girls with faster breast development had a higher BMI at around 14 to 18 years of age (Figure S4i), and girls with faster pubic hair development had a higher BMI throughout age 7 to 18 years (Figure S4l).

### Main analyses

**Figure 1:**
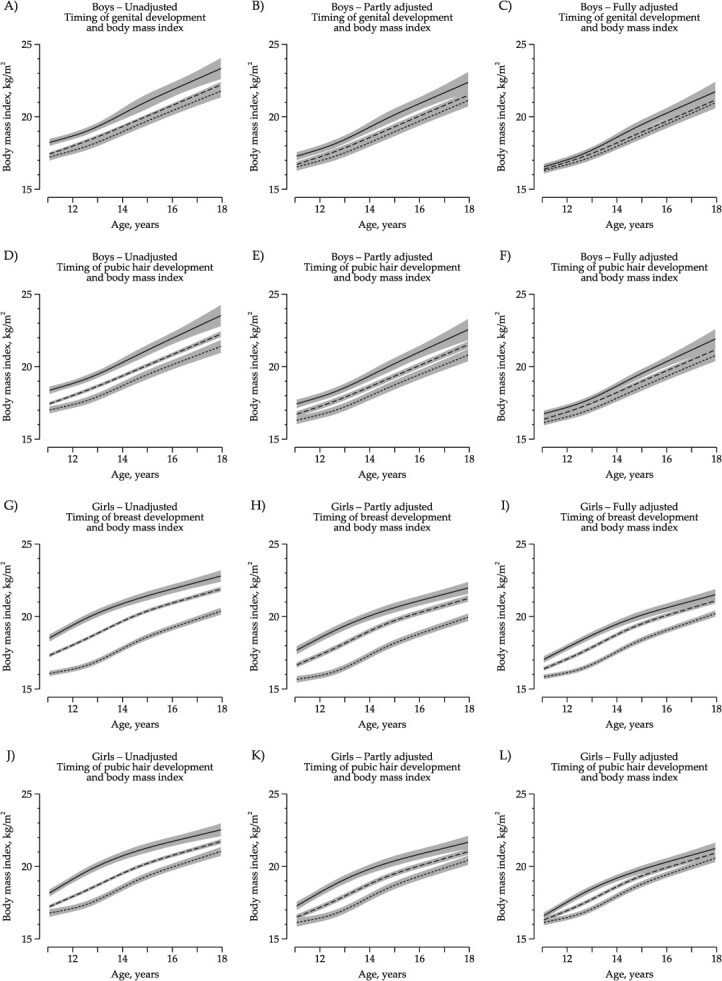
Main analysis of pubertal timing. BMI trajectories from 11 to 18 years according to pubertal timing unadjusted, fully adjusted for potential confounders, and further adjusted for childhood body mass index in 9520 children, the Puberty Cohort in the Danish National Birth Cohort, Denmark, 2000-2021. Solid line represents children with early pubertal timing, dashed line represents children with average pubertal timing, and short dashed line represents children with late pubertal timing. Shades correspond to 95% confidence intervals. (A-F) Boys. (G-L) Girls. (B, E, H, K) BMI trajectories presented for a reference person that had a birth weight of 3500 g and whose mother was a high-grade professional, had a prepregnant normal weight, did not smoke during the first trimester, and had menarche at the same time as her peers. (C, F, I, L) BMI trajectories presented for a reference person who had a birth weight of 3500 g and childhood BMI at 7 years of 15 kg/m^2^, whose mother was a high-grade professional, had prepregnant normal weight, did not smoke during the first trimester, and had menarche at the same time as her peers.

For each of the Tanner traits, children with earlier pubertal timing had a higher BMI trajectory, and children with later pubertal timing had a lower BMI trajectory from 11 to 18 years than children with average pubertal timing (Figure 1). The difference between BMI trajectories in boys attenuated after adjustment for potential confounders and was almost comparable after adjustment for childhood BMI at 7 years (Figure 1A-F). The difference between BMI trajectories in girls remained after adjustment for potential confounders, and weak associations remained after further adjustment for childhood BMI at 7 years (Figure 1G-L).

**Figure 2:**
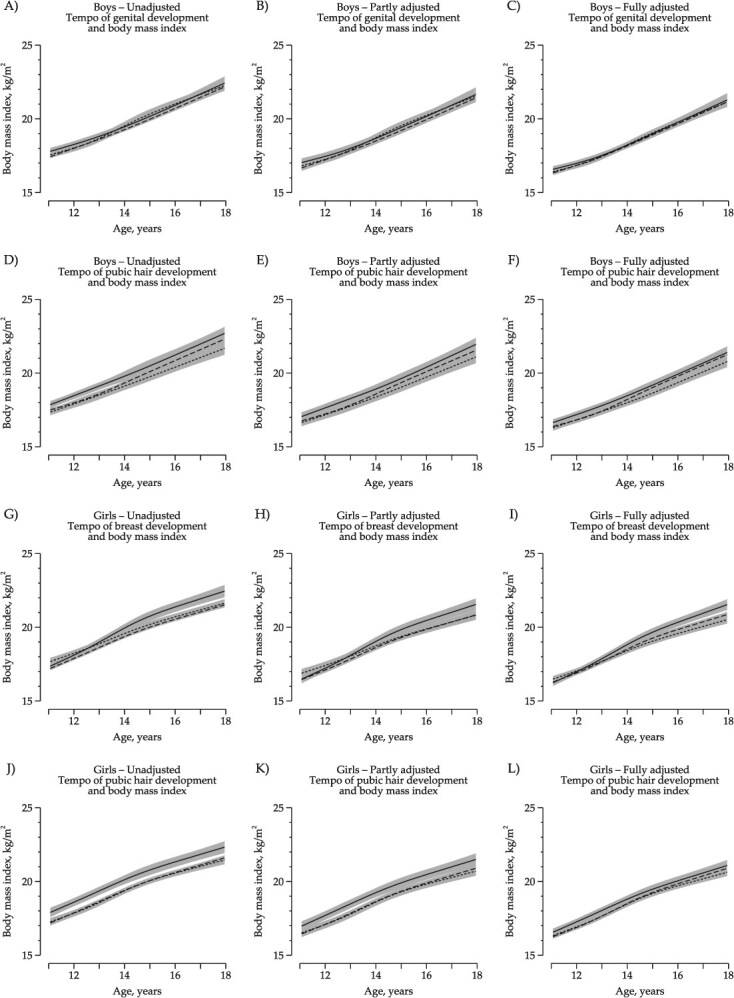
Main analysis of pubertal tempo. BMI trajectories from 11 to 18 years according to pubertal tempo unadjusted, fully adjusted for potential confounders, and further adjusted for childhood body mass index in 9520 children, the Puberty Cohort in the Danish National Birth Cohort, Denmark, 2000-2021. Solid line represents children with fast pubertal tempo, dashed line represents children with average pubertal tempo, and short dashed line represents children with slow pubertal tempo. Shades correspond to 95% confidence intervals. (A-F) Boys. (G-L) Girls. (B, E, H, K) BMI trajectories presented for a reference person who had a birth weight of 3500 g and whose mother was a high-grade professional, had a prepregnant normal weight, did not smoke during the first trimester, and had menarche at the same time as her peers. (C, F, I, L) BMI trajectories presented for a reference person who had a birth weight of 3500 g and childhood BMI at 7 years of 15 kg/m^2^, whose mother was a high-grade professional, had a prepregnant normal weight, did not smoke during the first trimester, and had menarche at the same time as her peers.

In boys, BMI trajectories did not differ across levels of pubertal tempo (Figure 2A-F). In girls, fast tempo of breast development was associated with a slightly higher BMI trajectory from around 16 to 18 years of age, even in the fully adjusted model (Figure 2G-I).

### Subanalyses

In the fully adjusted model, BMI at 18 years of age was 0.6 kg/m^2^ (95% CI, –0.2 to 1.4 kg/m^2^) and 0.7 kg/m^2^ (95% CI, –0.1 to 1.5 kg/m^2^) higher in young men with earlier timing of genital and pubic hair development, respectively, compared to young men with average pubertal timing. BMI was 0.4 kg/m^2^ (95% CI, 0.0-0.8 kg/m^2^) higher in young women with earlier pubertal timing of both breast and pubic hair development compared to young women with average pubertal timing. In the fully adjusted model, later pubertal timing was associated with lower BMI at 18 years in both men and women (Table 3).

**Table 3:** Subanalysis of pubertal timing and pubertal tempo, respectively, with BMI at 18 years (difference in kg/m^2^) according to pubertal timing and pubertal tempo unadjusted, partially adjusted for potential confounders, and fully adjusted including childhood BMI in 5381 children, the Puberty Cohort in the Danish National Birth Cohort, Denmark, 2000-2021.

	**Boys (difference in BMI in kg/m** ^**2**^ **)**					
	**Genital development**			**Pubic hair development**		
Pubertal timing	Early	Average	Late	Early	Average	Late
Crude	1.2 (0.3 to 2.1)	Ref	–0.4 (–0.9 to 0.2)	1.3 (0.4 to 2.2)	Ref	–0.8 (–1.3 to –0.3)
Partially adjusted^a^	0.9 (0.1 to 1.8)	Ref	–0.3 (–0.8 to 0.2)	1.1 (0.2 to 1.9)	Ref	–0.7 (–1.2 to –0.2)
Fully adjusted^b^	0.6 (–0.2 to 1.4)	Ref	0.1 (–0.6 to 0.3)	0.7 (–0.1 to 1.5)	Ref	–0.4 (–0.8 to 0.1)
						
Pubertal tempo	Fast	Average	Slow	Fast	Average	Slow
Crude	0.0 (–0.6 to 0.6)	Ref	0.1 (–0.5 to 0.6)	0.4 (–0.1 to 0.9)	Ref	–0.3 (–0.9 to 0.3)
Partially adjusted^a^	0.0 (–0.6 to 0.5)	Ref	0.1 (–0.4 to 0.6)	0.5 (0.0 to 1.0)	Ref	–0.2 (–0.7 to 0.4)
Fully adjusted^b^	–0.1 (–0.7 to 0.4)	Ref	0.0 (–0.5 to 0.5)	0.3 (–0.2 to 0.8)	Ref	–0.2 (–0.7 to 0.3)
	**Girls (difference in BMI in kg/m** ^**2**^ **)**					
	**Breast development**			**Pubic hair development**		
Pubertal timing	Early	Average	Late	Early	Average	Late
Crude	1.1 (0.7 to 1.5)	Ref	–1.8 (–2.2 to –1.5)	1.0 (0.5 to 1.5)	Ref	–1.0 (–1.4 to –0.6)
Partially adjusted^a^	0.9 (0.5 to 1.3)	Ref	–1.6 (–1.9 to –1.2)	0.8 (0.3 to 1.2)	Ref	–0.8 (–1.2 to –0.5)
Fully adjusted^b^	0.4 (0.0 to 0.8)	Ref	–1.1 (–1.4 to –0.8)	0.4 (0.0 to 0.8)	Ref	–0.5 (–0.8 to –0.2)
						
Pubertal tempo	Fast	Average	Slow	Fast	Average	Slow
Crude	0.6 (0.2 to 1.0)	Ref	–0.3 (–0.7 to 0.0)	0.8 (0.3 to 1.3)	Ref	–0.4 (–0.8 to 0.0)
Partially adjusted^a^	0.5 (0.1 to 0.9)	Ref	–0.4 (–0.7 to 0.0)	0.6 (0.1 to 1.1)	Ref	–0.4 (–0.8 to 0.0)
Fully adjusted^a^	0.4 (0.0 to 0.7)	Ref	–0.6 (–0.9 to –0.3)	0.2 (–0.2 to 0.7)	Ref	–0.4 (–0.7 to 0.0)

Abbreviation: BMI, body mass index.

a Adjusted for highest educational level of the parents, maternal age at menarche, maternal prepregnancy BMI, maternal smoking in first trimester, and birth weight.

b Adjusted for highest educational level of the parents, maternal age at menarche, maternal prepregnancy BMI, maternal smoking in first trimester, birth weight, and childhood BMI.

Pubertal tempo was not as strongly associated with BMI at 18 years in men (Table 3). In the fully adjusted model, slow tempo of breast and pubic hair development in women was associated with lower BMI at 18 years of age (–0.6 kg/m^2^ [95% CI, –0.9 to –0.3 kg/m^2^] and –0.4 kg/m^2^ [95% CI, –0.7 to 0.0 kg/m^2^], respectively) (Table 3).

In the fully adjusted joint model, women with early timing and fast tempo of breast development had a higher BMI at 18 years of 1.1 kg/m^2^ (95% CI 0.3 to 1.9 kg/m^2^), and women with late timing and slow tempo of breast development had a lower BMI of –1.1 kg/m^2^ (95% CI, –1.9 to –0.3 kg/m^2^) compared with women with average timing and tempo. Moreover, late timing and slow tempo of pubic hair development in men were associated with –0.9 kg/m^2^ (95% CI, –1.5 to –0.2 kg/m^2^) lower BMI compared with men with average pubertal timing and tempo (Table 4).

**Table 4:** Subanalysis of the interaction between pubertal timing and tempo, with BMI at 18 years (difference in kg/m^2^) according to both pubertal timing and pubertal tempo unadjusted, partially adjusted for potential confounders, and fully adjusted including childhood BMI in 5381 children, the Puberty Cohort in the Danish National Birth Cohort, Denmark, 2000-2021.

	**Early timing**			**Average timing**			**Late timing**		
**Difference in BMI, kg/m** ^**2**^	**Crude**	**Partially adjusted** ^**a**^	**Fully adjusted** ^**b**^	**Crude**	**Partially adjusted** ^**a**^	**Fully adjusted** ^**b**^	**Crude**	**Partially adjusted** ^**a**^	**Fully adjusted** ^**b**^
Genital development in boys^c^									
Fast tempo	1.1 (0.0 to 2.3)	1.0 (–0.1 to 2.2)	0.3 (–0.8 to 1.4)	–0.2 (–0.8 to 0.5)	–0.2 (–0.8 to 0.4)	–0.2 (–0.9 to 0.4)	0.2 (–1.4 to 1.7)	0.1 (–1.3 to 1.6)	0.7 (–0.6 to 2.1)
Average tempo	1.2 (–0.1 to 2.6)	0.9 (–0.4 to 2.2)	0.5 (–0.7 to 1.8)	Ref			–0.3 (–1.0 to 0.3)	–0.3 (–0.9 to 0.3)	–0.1 (–0.7 to 0.4)
Slow tempo	1.0 (–0.1 to 2.1)	1.0 (–0.1 to 2.1)	0.9 (–0.2 to 1.9)	0.3 (–0.4 to 0.9)	0.3 (–0.4 to 0.9)	0.1 (–0.5 to 0.7)	–0.5 (–1.4 to 0.3)	–0.5 (–1.4 to 0.4)	–0.4 (–1.1 to 0.3)
Pubic hair development in boys^d^									
Fast tempo	1.2 (–0.1 to 2.6)	1.0 (–0.3 to 2.4)	0.5 (–0.9 to 1.9)	0.5 (0.0 to 1.0)	0.5 (0.0 to 1.0)	0.4 (–0.1 to 0.9)	–0.3 (–1.9 to 1.3)	–0.1 (–1.6 to 1.4)	0.4 (–1.0 to 1.9)
Average tempo	1.6 (0.4 to 2.8)	1.3 (0.2 to 2.4)	0.9 (–0.1 to 2.0)	Ref			–0.5 (–1.2 to 0.1)	–0.4 (–1.0 to 0.3)	0.0 (–0.6 to 0.6)
Slow tempo	0.7 (–0.6 to 2.1)	0.8 (–0.4 to 2.1)	0.7 (–0.4 to 1.8)	0.3 (–0.5 to 1.0)	0.4 (–0.4 to 1.1)	0.3 (–0.4 to 1.0)	–1.1 (–1.9 to –0.2)	–1.0 (–1.7 to –0.2)	–0.9 (–1.5 to –0.2)
Breast development in girls^e^									
Fast tempo	2.0 (1.1 to 2.8)	1.5 (0.7 to 2.4)	1.1 (0.3 to 1.9)	–0.1 (–0.6 to 0.4)	–0.1 (–0.6 to 0.4)	–0.1 (–0.5 to 0.4)	–1.1 (–2.0 to –0.3)	–0.8 (–1.6 to 0.0)	–0.5 (–1.1 to 0.1)
Average tempo	1.0 (0.4 to 1.6)	0.9 (0.3 to 1.4)	0.4 (–0.1 to 0.9)	Ref			–2.1 (–2.5 to –1.8)	–1.8 (–2.2 to –1.4)	–1.4 (–1.7 to –1.0)
Slow tempo	0.1 (–0.4 to 0.7)	–0.1 (–0.6 to 0.5)	–0.6 (–1.1 to –0.1)	–0.7 (–1.2 to –0.2)	–0.7 (–1.1 to –0.2)	–0.8 (–1.2 to –0.4)	–1.5 (–2.4 to –0.6)	–1.4 (–2.2 to –0.6)	–1.1 (–1.9 to –0.3)
Pubic hair development in girls^f^									
Fast tempo	0.6 (0.0 to 1.2)	0.4 (–0.2 to 1.0)	–0.1 (–0.6 to 0.4)	1.1 (0.3 to 1.8)	0.9 (0.2 to 1.6)	0.6 (0.0 to 1.2)	–1.3 (–2.2 to –0.4)	–1.0 (–1.9 to –0.1)	–0.8 (–1.6 to –0.1)
Average tempo	1.3 (0.7 to 1.9)	1.0 (0.4 to 1.6)	0.6 (0.1 to 1.1)	Ref			–0.8 (–1.2 to –0.3)	–0.6 (–1.1 to –0.2)	–0.4 (–0.8 to 0.0)
Slow tempo	1.7 (–0.5 to 3.9)	1.6 (–0.6 to 3.8)	1.2 (–0.9 to 3.4)	–0.4 (–0.8 to 0.1)	0.4 (–0.8 to 0.0)	–0.4 (0.8 to –0.1)	–1.1 (–1.6 to –0.6)	–1.0 (–1.6 to –0.4)	–0.7 (–1.2 to –0.2)

Abbreviations: BMI, body mass index.

a Adjusted for highest educational level of the parents, maternal age at menarche, maternal prepregnancy BMI, maternal smoking in first trimester, and birth weight.

b Adjusted for highest educational level of the parents, maternal age at menarche, maternal prepregnancy BMI, maternal smoking in first trimester, birth weight, and childhood BMI.

c No statistically significant interaction between pubertal timing and tempo in the association with BMI (*P* = .5).

d Statistically significant interaction between pubertal timing and tempo in the association with BMI (*P* = .04).

e Statistically significant interaction between pubertal timing and tempo in the association with BMI (*P* < .001).

f Statistically significant interaction between pubertal timing and tempo in the association with BMI (*P* < .001).

## Discussion

### Key results

Earlier pubertal timing was more strongly associated with higher BMI trajectories than faster pubertal tempo. Participants with earlier pubertal timing had a higher BMI before, during, and after puberty compared to participants with average pubertal timing. However, in boys, the associations between pubertal timing and BMI trajectories virtually disappeared after adjustment for potential confounding factors and childhood BMI, suggesting that the higher BMI trajectories observed in boys with earlier pubertal timing were largely explained by a higher childhood BMI. In girls, the association remained, albeit not as strong as after adjustment for childhood BMI, suggesting that the higher BMI trajectories observed in girls with earlier pubertal timing were not solely due to a higher childhood BMI. Pubertal tempo was associated with a slightly higher BMI in young adulthood in women only. Overall, we found similar results in the subanalyses when investigating associations between pubertal timing and tempo and adult BMI separately. When investigating the joint effect of both pubertal timing and tempo on adult BMI, the associations were even more pronounced.

### Strengths and limitations

The main strength of this study was the large study sample from the general Danish population, with multiple measures on both pubertal timing and tempo and BMI derived from measures of weight and height from preventive health visits among health nurses in school settings and the DNBC. Moreover, we had detailed information on potential confounding factors, including the information on childhood BMI.

The percentage of children included in the main analysis investigating BMI trajectories was 57%, and only 24% were included in the subanalyses investigating adult BMI. As most potential confounding factors, including childhood BMI, were associated with participation in the Puberty Cohort (Table S3), we accounted for this by applying inverse probability of selection weights in all analyses. Further, in a previous study, we found minimal risk of selection bias in the Puberty Cohort since a marker of pubertal timing—the height difference in standard deviations—was not associated with participation in the Puberty Cohort.^26^ Further, if children or adolescents with a higher BMI tended not to participate, this would most likely not explain our findings; rather, it would cause bias toward the null. We, therefore, consider the risk of selection bias to be modest at most.

We relied on self-reported measures of pubertal timing and tempo, which allowed for the collection of puberty information from a large cohort with a high participation rate. This increases power and limits the risk of selection bias. This, however, comes with an increased risk of bias due to misclassification of pubertal timing and tempo. The participants returned questionnaires on current pubertal development throughout pubertal maturation, limiting the risk of misclassification due to recall. Still, it may be difficult for some children to accurately distinguish between the Tanner stages. The agreement between self-assessment and an expert evaluation has, however, been found to be moderate to good in populations similar to our study population,^27^ and the validity of the self-assessment in the puberty cohort has also been found to be fair to moderate.^28^ Any misclassification has been found to be nondependent on parental education and is expected to be nondifferential regarding the remaining covariates and BMI. One exception, however, is that the Tanner staging of breast development may be prone to differential misclassification since girls with obesity could mistake adipose tissue for breast tissue.^29^ This might explain the association between the timing and tempo of breast development and the BMI trajectories.

The height and weight measures obtained from the Children’s Database were done by school nurses or general practitioners and were therefore objectively measured in accordance with guidelines. Contrary, in the DNBC, height and weight measures were obtained by self-report and with some lag time. This may induce some measurement errors, most likely of a nondifferential nature, causing bias toward the null, and therefore cannot explain the observed associations between pubertal development and BMI trajectories.

Even though we adjusted for multiple potential confounding factors, we cannot eliminate the risk of unmeasured or residual confounding due to the observational design employed in the study.

Some girls (85%) and boys (65%) had already entered puberty (Tanner stage 2) at 11 years, when the data collection on pubertal development started. Hence, we were not able to derive the timing and tempo at the onset of puberty but had to derive timing and tempo midway through puberty (Tanner stage 3), when we had good data coverage.

### Interpretation

Our results indicate that boys with earlier pubertal timing may experience a higher BMI due to a higher childhood BMI. Pubertal timing in relation to higher BMI in young adult men has previously been investigated in terms of the timing of facial hair and body hair,^30^ voice break,^30^^,^^31^ Tanner stages,^9^^,^^32^ and age at peak height velocity (aPHV).^33^^-^^35^ All but 1 study^9^ found that earlier pubertal timing was associated with a higher adult BMI.^30^^-^^35^ However, after adjustment for childhood BMI, the associations disappeared in some studies,^31^^,^^32^^,^^35^ whereas the associations only slightly attenuated and remained in other studies.^30^^,^^33^^,^^34^ Therefore, it remains to be fully elucidated whether earlier pubertal timing in boys is associated with higher adult BMI independent of childhood BMI.

In girls, earlier pubertal timing may have an independent effect on higher BMI regardless of childhood BMI. Pubertal timing in relation to higher BMI in young adult women has previously been investigated mostly in terms of age at menarche,^9^^,^^30^^,^^31^^,^^36^^-^^41^ but Tanner stages^9^^,^^32^ and aPHV^33^^-^^35^ have also been investigated. Earlier age at menarche was associated with higher BMI in adulthood in all studies,^9^^,^^30^^,^^31^^,^^36^^-^^40^ except 1 study.^41^ When considering childhood BMI, most studies found that the associations remained but attenuated somewhat,^9^^,^^30^^,^^31^^,^^38-40^ like the findings in our study. In a recent meta-analysis of genetic studies, the author concluded that higher childhood BMI was causally related to earlier age at menarche, which in turn was causally related to higher adult BMI.^42^ In contrast, results have been inconsistent for Tanner stages, with 1 study finding no associations between Tanner stages at age 12 years and BMI in young adulthood,^9^ and 1 study finding an association that attenuated with further adjustment for childhood BMI.^32^ Similarly, aPHV has been inconsistently associated with higher BMI^33^^-^^35^ (Table S1). Overall, it is still inconclusive whether different markers of pubertal timing in girls are associated with higher adult BMI independent of childhood BMI.

Only 1 previous study has investigated the association between pubertal tempo—measured using latent class growth analysis of the 4 Tanner traits—and higher BMI in young adulthood.^9^ Contrary to our study, that study investigating a sample of primarily Black men and women from South Africa found that fast progression through puberty was associated with a higher BMI at 18 to 20 years in both men and women, but the estimates attenuated after adjustment for childhood BMI.^9^ Pubertal tempo was not strongly associated with adult BMI in our study. However, the interaction analyses showed indications that fast pubertal tempo was associated with a higher adult BMI in men and women experiencing earlier pubertal timing. Moreover, slow pubertal tempo was associated with a lower adult BMI in men and women experiencing later pubertal timing. Further research on the potential association between pubertal tempo and BMI and the possible interaction with pubertal timing is needed to make firm conclusions.

In conclusion, we found that the associations between earlier pubertal timing and adult BMI in young men were largely driven by childhood BMI, indicating that boys with earlier pubertal timing may experience a higher BMI due to a higher childhood BMI. In contrast, earlier pubertal timing appeared to result in a higher adult BMI in young women, even after adjustment for childhood BMI, indicating that early pubertal timing may have an independent effect on higher BMI regardless of childhood BMI. A faster pubertal tempo appeared to result in a slightly higher BMI in young adult women only.

## Ethics

This study was conducted in accordance with the Declaration of Helsinki. The Committee for Biomedical Research Ethics in Denmark approved the data collection in the DNBC ((KF)01-471/94). A written informed consent was obtained from the mother upon recruitment, including permission to follow up until the child turned 18 years of age. The present study was approved by the Danish Data Protection Agency (2012-41-0379 and 2015-57-0002) and the Steering Committee of the DNBC (2012-04, 2015-47, and 2018-27). The data were deidentified by removing personal identifiers; hence, no protocol approval from an institutional review board was required.

## Supplementary Material

Web_Material_kwaf063

## Data Availability

The data are not publicly available due to local regulations (the General Data Protection Regulation [GDPR]). However, researchers may apply for access to data from the DNBC. Please see https://www.dnbc.dk/data-available or write to dnbc-research@ssi.dk for additional information.
