# Supplementary material for: Pubertal timing and tempo and body mass index trajectories: investigating the confounding role of childhood body mass index
Source: Am J Epidemiol. 2025 Mar 21;194(8):2270–80. doi: 10.1093/aje/kwaf063 (PMC12342948; doi:10.1093/aje/kwaf063)
Supplement: Web_Material_kwaf063 [file web_material_kwaf063.docx]

**Supplementary information**

**Article title: Pubertal timing and tempo and body mass index trajectories: investigating the confounding role of childhood body mass index**

**Journal: American Journal of Epidemiology**

**Authors: Anne Gaml-Sørensen, Nis Brix, Andreas Ernst, Lea Lykke Harrits Lunddorf, Onyebuchi A. Arah,** **Katrine Strandberg-Larsen, Cecilia Høst Ramlau-Hansen**

**Correspondance: Anne Gaml-Sørensen, Bartholins Allé 2, Aarhus University, 8000 Aarhus C, Denmark. Tel: +45 40868183; E-mail: ags@ph.au.dk ; ORCiD iD: 0000-0002-5242-939X**

**Page 2: TABLE S1: Overview of existing literature.**

**Page 8: FIGURE S1: Flowchart of the inclusion of the study population**

**Page 9: SUPPLEMENTARY FIGURE 2: Directed acyclic graph**

**Page 10: SUPPLEMENTARY FIGURE 3: Descriptive analysis of pubertal timing**

**Page 12: SUPPLEMENTARY FIGURE 4: Descriptive analysis of pubertal tempo**

**Page 14: SUPPLEMENTARY TABLE 2: Total number of completed questionnaires in the Puberty Cohort**

**Page 15: SUPPLEMENTARY TABLE 3: Baseline characteristics according to participation in the Puberty Cohort**

**SUPPLEMENTARY TABLE 1: Overview of existing literature.**

**Follow-up studies examining the association between pubertal timing and different measures of post-pubertal obesity or adiposity with or without taking the potential confounding effect of increased childhood body mass index (BMI) into account. This overview only includes studies published after the systematic review and meta-analysis by Prentice and Viner.^1^**

| **Title** | **First Author & year** | **Country** | **Study population** | **Exposure** | **Outcome** | **Adjustment for childhood BMI** | **Conclusion** |
| --- | --- | --- | --- | --- | --- | --- | --- |
| Adolescent and young adult female determinants of visceral adipose tissue at ages 26-28 years^2^ | Charles J Glueck, 2015 | USA | N = 369 black and white girls | Age at menarche | Visceral adipose tissue assessed by magnetic resonance imaging | No | Earlier age at menarche was associated with greater visceral adipose tissue at age 26-28 years. |
| Age at Menarche and Cardiometabolic Risk in Adulthood: The Coronary Artery Risk Development in Young Adults Study^3^ | Jill Dreyfus, 2015 | USA | N = 1,333 African American and n = 1,250 White women | Age at menarche | BMI and waist circumference (WC) | No | Earlier age at menarche was associated with higher BMI and increased WC at age 42-59 years. |
| Pubertal Development and Prepubertal Height and Weight Jointly Predict Young Adult Height and Body Mass Index in a Prospective Study in South Africa^4^ | Aryeh D Stein, 2016 | South Africa | N = 823 mostly black women and n = 765 mostly black men | Pubertal status and pubertal progression based on clinically assessed and self-reported Tanner Stages  Age at menarche | WHO defined overweight and obesity | Yes | Fast progression through puberty was associated with an increased risk of overweight and obesity at 18-20 years in both women and men, but the estimates attenuated after adjustment for childhood BMI.  Earlier pubertal status at 12 years was not associated with risk of overweight or obesity at 18-20 years in either women or men.  Earlier age at menarche was associated with a higher risk of overweight or obesity, but the estimates attenuated after adjustment for childhood BMI. |
| Impact of the age at menarche on body composition in adulthood: results from two birth cohort studies^5^ | Susana Bubach, 2016 | Brazil | N = 1,637 and n = 2,033 women born in 1982 and 1993, respectively | Age at menarche | BMI and body composition at age 18 years and age 30 years, respectively | Yes | Early menarche was associated with higher BMI, WC, abdominal visceral fat layer thickness, fat mass index, fat-free mass index and prevalence of overweight at age 18 years and 30 years, respectively.  After adjustment for childhood BMI, associations attenuated. |
| Influence of puberty timing on adiposity and cardiometabolic traits: A Mendelian randomisation study^6^ | Joshua A Bell, 2018 | United Kingdom | N = 2,112 white-European women and n = 1,499 white-European men from the Avon Longitudinal Study of Parents and Children (ALSPAC) cohort | Age at menarche  Age at voice break | BMI and body composition | Yes | Early menarche and earlier age at voice break was associated with higher BMI and higher fat mass index at 18 years in ordinary observational analyses.  After adjustment for childhood BMI, associations attenuated.  In the Mendelian Randomisation analyses, genetically predicted earlier age at menarche was associated with higher BMI and higher fat mass index at 18 years.  After adjustment for childhood BMI, associations attenuated. |
| Age at menarche and adult body mass index: a Mendelian randomization study^7^ | Dipender Gill, 2018 | United Kingdom | N = 70,692 women from the UK Biobank and the genetic investigation of anthropometric traits (GIANT) Consortium | Age at menarche | BMI | No/yes  Exclusion of genetic instruments affecting childhood BMI. | Genetically predicted earlier age at menarche was associated with higher adult BMI. When excluding genetic instruments also affecting childhood BMI the associations between age at menarche and adult BMI remained, albeit it attenuated somewhat. |
| Age at menarche and childhood body mass index as predictors of cardio-metabolic risk in young adulthood: A prospective cohort study^8^ | Chi Le-Ha, 2018 | Australia | N = 650 women (85% Caucasian) | Age at menarche | BMI | Yes | Early menarche was associated with higher BMI at age 17 and 20 years.  After adjustment for childhood BMI, associations attenuated. |
| Timing of puberty in boys and girls: Implications for population health^9^ | Lindsay T Hoyt, 2020 | USA | N = 7,728 women and n = 6,817 men from Add Health, a nationally representative sample | Age at menarche  Age at facial hair, body hair, and voice change  Peer-relative pubertal timing (subjective comparison of advancement in pubertal development relative to peers) | BMI | No/yes.  Adjustment for adolescence BMI at age 12-19 years | Early menarche and early facial hair, body hair and voice change were associated with higher BMI at age 24-32.  Adjustment for adolescence BMI at age 12-19 years attenuated the results in boys but results remained significant in girls. |
| Puberty timing and adiposity change across childhood and adolescence: disentangling cause and consequence^10^ | Linda M O'Keeffe, 2020 | United Kingdom | N = 4,176 boys and girls from the ALSPAC cohort | Age at peak height velocity (aPHV) at 5-20 years of age | Fat mass trajectories with 18 232 repeated measures of fat mass from age 9-18 years. | Yes | A 1-year later aPHV was associated with lower fat mass in girls and boys at 9 years. These differences were smaller at age 18 years.  Trajectories provided evidence that pre-pubertal fat mass was strongly associated with puberty timing, and little evidence of an association of puberty timing with post-pubertal fat mass change. |
| Causal relationship between the timing of menarche and young adult body mass index with consideration to a trend of consistently decreasing age at menarche^11^ | Hakyung Kim, 2021 | Korea | N = 4,093 women | Age at menarche | BMI | No | Earlier age at menarche is not strongly associated with higher BMI in adulthood. |
| The Association Between Puberty Timing and Body Mass Index in a Longitudinal Setting: The Contribution of Genetic Factors^12^ | Karri Silventoinen, 2022 | Finland | N = 9,080 twins (twin design) and n = 2,468 twins (genetic analysis) | The pubertal developmental scale. Polygenetic risk scores for BMI, waist-to-hip ratio adjusted for BMI and WC | BMI | Yes | Early puberty timing was associated with higher BMI in adulthood mostly in girls and slightly in boys. Correlations were weak and largely disappeared after adjusting for childhood BMI.  Early puberty is not an independent risk factor for adult obesity but rather reflects the association between puberty timing and childhood BMI contributed by genetic predisposition. |
| Association between height growth patterns in puberty and stature in late adolescence: A longitudinal analysis in chinese children and adolescents from 2006 to 2016^13^ | Li Chen, 2022 | China | N = 13,143 boys and girls from Zhongshan city | aPHV, peak height velocity (PHV), and age at take-off (TOA) | Height, overweight and obesity at 18 years of age | Yes | Earlier aPHV was associated with overweight and obesity at 18 years of age.  Pubertal growth patterns, including earlier puberty timing, smaller puberty intensity, and shorter puberty spurt duration, was associated with higher overweight and obesity risks in late adolescence. |
| Prepubertal BMI, pubertal growth patterns, and long-term BMI: Results from a longitudinal analysis in Chinese children and adolescents from 2005 to 2016^14^ | Yanhui Li, 2022 | China | N = 9606 boys and girls from Zhongshan city | aPHV and PHV | BMI | Yes | Earlier aPHV was associated with higher risk of overweight and obesity at 18 years of age.  Higher PHV was associated with higher risk of long-term underweight in girls. |

Abbreviations: Body mass index: BMI; age at peak height velocity: aPHV; peak height velocity: PHV; waist circumference: WC

**References**

1. Prentice P, Viner RM. Pubertal timing and adult obesity and cardiometabolic risk in women and men: a systematic review and meta-analysis. *Int J Obes (Lond)* 2013; **37**: 1036-43.

2. Glueck CJ, Wang P, Woo JG, Morrison JA, Khoury PR, Daniels SR. Adolescent and young adult female determinants of visceral adipose tissue at ages 26-28 years. *J Pediatr* 2015; **166**: 936-46.e1-3.

3. Dreyfus J, Jacobs DR, Jr., Mueller N, et al. Age at Menarche and Cardiometabolic Risk in Adulthood: The Coronary Artery Risk Development in Young Adults Study. *J Pediatr* 2015; **167**: 344-52.e1.

4. Stein AD, Lundeen EA, Martorell R, et al. Pubertal Development and Prepubertal Height and Weight Jointly Predict Young Adult Height and Body Mass Index in a Prospective Study in South Africa. *J Nutr* 2016; **146**: 1394-401.

5. Bubach S, Menezes AM, Barros FC, et al. Impact of the age at menarche on body composition in adulthood: results from two birth cohort studies. *BMC Public Health* 2016; **16**: 1007.

6. Bell JA, Carslake D, Wade KH, et al. Influence of puberty timing on adiposity and cardiometabolic traits: A Mendelian randomisation study. *PLoS Med* 2018; **15**: e1002641.

7. Gill D, Brewer CF, Del Greco MF, et al. Age at menarche and adult body mass index: a Mendelian randomization study. *Int J Obes (Lond)* 2018; **42**: 1574-81.

8. Le-Ha C, Beilin LJ, Burrows S, et al. Age at menarche and childhood body mass index as predictors of cardio-metabolic risk in young adulthood: A prospective cohort study. *PLoS One* 2018; **13**: e0209355.

9. Hoyt LT, Niu L, Pachucki MC, Chaku N. Timing of puberty in boys and girls: Implications for population health. *SSM Popul Health* 2020; **10**: 100549.

10. O'Keeffe LM, Frysz M, Bell JA, Howe LD, Fraser A. Puberty timing and adiposity change across childhood and adolescence: disentangling cause and consequence. *Hum Reprod* 2020; **35**: 2784-92.

11. Kim H, Choe SA, Lee SJ, Sung J. Causal relationship between the timing of menarche and young adult body mass index with consideration to a trend of consistently decreasing age at menarche. *PLoS One* 2021; **16**: e0247757.

12. Silventoinen K, Jelenkovic A, Palviainen T, Dunkel L, Kaprio J. The Association Between Puberty Timing and Body Mass Index in a Longitudinal Setting: The Contribution of Genetic Factors. *Behav Genet* 2022; **52**: 186-94.

13. Chen L, Su B, Zhang Y, et al. Association between height growth patterns in puberty and stature in late adolescence: A longitudinal analysis in chinese children and adolescents from 2006 to 2016. *Front Endocrinol (Lausanne)* 2022; **13**: 882840.

14. Li Y, Gao D, Liu J, et al. Prepubertal BMI, pubertal growth patterns, and long-term BMI: Results from a longitudinal analysis in Chinese children and adolescents from 2005 to 2016. *Eur J Clin Nutr* 2022; **76**: 1432-9.

**Figure S1: Flowchart**

Flow diagram of the inclusion of children included in the analysis, the Puberty Cohort within the Danish National Birth Cohort, Denmark, 2000-2021.

Abbreviation: DNBC, Danish National Birth Cohort. BMI, Body mass index.



**Figure S2: Directed acyclic graph**

Directed acyclic graph (DAG) illustrating the assumed causal framework of the study on pubertal timing and tempo and BMI trajectories. Boxes indicate conditioning in the statistical analyses.

Abbreviation: BMI, Body mass index.

**SUPPLEMENTARY FIGURE 3: Descriptive analysis of pubertal timing**

Height, weight, and body mass index trajectories from 7 to 18 years according to pubertal timing in 12 812 children, the Puberty Cohort in the Danish National Birth Cohort, Denmark, 2000-2021. Solid line represents children with early pubertal timing, dashed line represents children with average pubertal timing, and short dashed line represents children with late pubertal timing. Shades correspond to 95% confidence intervals. Panel a.–f.: Boys. Panel g.–l.: Girls.

**SUPPLEMENTARY FIGURE 4: Descriptive analysis of pubertal tempo**

Height, weight, and body mass index trajectories from 7 to 18 years according to pubertal tempo in 12 812 children, the Puberty Cohort in the Danish National Birth Cohort, Denmark, 2000-2021. Solid line represents children with fast pubertal tempo, dashed line represents children with average pubertal tempo, and short dashed line represents children with slow pubertal tempo. Shades correspond to 95% confidence intervals. Panel a.–f.: Boys. Panel g.–l.: Girls.

| Supplementary Table 2. Total number of completed questionnaires in the Puberty Cohort, the Danish National Birth Cohort, Denmark, 2012–2022. | |  |
| --- | --- | --- |
|  |  |  |
| Number of completed puberty questionnaires | n (%) |  |
| 1 | 2600 (16.4) |  |
| 2 | 1177 (7.4) |  |
| 3 | 1143 (7.2) |  |
| 4 | 1096 (6.9) |  |
| 5 | 1137 (7.2) |  |
| 6 | 1307 (8.3) |  |
| 7 | 1326 (8.4) |  |
| 8 | 1312 (8.3) |  |
| 9 | 1160 (7.3) |  |
| 10 | 946 (6.0) |  |
| 11 | 788 (5.0) |  |
| 12 | 759 (4.8) |  |
| 13 | 668 (4.2) |  |
| 14 | 380 (2.4) |  |
| 15 | 20 (0.1) |  |
| In total | 15819 (100.0) |  |

| Supplementary Table 3. Baseline characteristics according to participation in the Puberty Cohort, the Danish National Birth Cohort, Denmark, 2000–2022. | | | | |  |
| --- | --- | --- | --- | --- | --- |
|  |  |  |  |  |  |
|  | Participation in the Puberty Cohort | | |  |  |
| Pubertal timing | Once | At least twice | Total | Missing |  |
| n (%) | 2600 (16.4) | 13219 (83.6) | 15819 (100.0) | 0% |  |
| Highest educational level of the parents | |  |  | 0.2% |  |
| High grade professional | 544 (20.9) | 3144 (23.8) | 3688 (23.4) |  |  |
| Low grade professional | 725 (27.9) | 4470 (33.9) | 5195 (32.9) |  |  |
| Skilled worker | <784^a^ (<30.2) | 3572 (27.1) | 4353 (27.6) |  |  |
| Unskilled worker | 477 (18.4) | 1672 (12.7) | 2149 (13.6) |  |  |
| Student and economically inactive | 70 (2.7) | 333 (2.5) | 403 (2.5) |  |  |
| Maternal age at menarche |  |  |  | 0.8% |  |
| Earlier than peers | 668 (25.9) | 3343 (25.5) | 4011 (25.6) |  |  |
| Same time as peers | 1476 (57.2) | 7512 (57.3) | 8988 (57.3) |  |  |
| Later than peers | 437 (16.9) | 2260 (17.2) | 2697 (17.2) |  |  |
| Maternal pre-pregnancy BMI |  |  |  | 1.5% |  |
| <18.5 | 187 (7.3) | 869 (6.7) | 1056 (6.8) |  |  |
| 18.5 - <25 | 1506 (58.5) | 8150 (62.6) | 9656 (61.9) |  |  |
| 25 - <30 | 601 (23.3) | 2704 (20.8) | 3305 (21.2) |  |  |
| 30+ | 281 (10.9) | 1304 (10.0) | 1585 (10.2) |  |  |
| Maternal smoking in 1st trimester |  |  |  | 0.3% |  |
| Non-smoker | 1762 (68.1) | 9585 (72.7) | 11347 (72.0) |  |  |
| -10 cigarettes/day | 634 (24.5) | 2878 (21.8) | 3512 (22.3) |  |  |
| >10 cigarettes/day | 193 (7.5) | 714 (5.4) | 907 (5.8) |  |  |
| Birth weight (kilogram)^b^ | 3542 (592) | 3526 (594) | 3529 (593) | 0.4% |  |
| Childhood BMI at 7 years^b^ | 15.8 (1.8) | 15.6 (1.7) | 15.6 (1.7) | 26% |  |
| Due to rounding of percentages, numbers may not add up to 100% | |  |  |  |  |
| Abbreviations: BMI, body mass index; SD, standard deviation | |  |  |  |  |
| a It is not allowed due to local data regulations to report smaller numbers than five, including missing data. Therefore, some numbers have been rounded up or down to mask the numbers smaller than five | | | | |  |
| b Mean (SD) |  |  |  |  |  |
